# SCINOBO: a novel system classifying scholarly communication in a dynamically constructed hierarchical Field-of-Science taxonomy

**DOI:** 10.3389/frma.2023.1149834

**Published:** 2023-05-04

**Authors:** Sotiris Kotitsas, Dimitris Pappas, Natalia Manola, Haris Papageorgiou

**Affiliations:** ^1^Athena Research Center, Institute for Language and Speech Processing, Athens, Greece; ^2^OpenAIRE, Athens, Greece

**Keywords:** field of science publication classification, multilayer network, Field of Science taxonomy, digital libraries, scholarly literature, natural language processing

## Abstract

Classifying scientific publications according to Field-of-Science taxonomies is of crucial importance, powering a wealth of relevant applications including Search Engines, Tools for Scientific Literature, Recommendation Systems, and Science Monitoring. Furthermore, it allows funders, publishers, scholars, companies, and other stakeholders to organize scientific literature more effectively, calculate impact indicators along Science Impact pathways and identify emerging topics that can also facilitate Science, Technology, and Innovation policy-making. As a result, existing classification schemes for scientific publications underpin a large area of research evaluation with several classification schemes currently in use. However, many existing schemes are domain-specific, comprised of few levels of granularity, and require continuous manual work, making it hard to follow the rapidly evolving landscape of science as new research topics emerge. Based on our previous work of scinobo, which incorporates metadata and graph-based publication bibliometric information to assign Field-of-Science fields to scientific publications, we propose a novel hybrid approach by further employing Neural Topic Modeling and Community Detection techniques to dynamically construct a Field-of-Science taxonomy used as the backbone in automatic publication-level Field-of-Science classifiers. Our proposed Field-of-Science taxonomy is based on the OECD fields of research and development (FORD) classification, developed in the framework of the Frascati Manual containing knowledge domains in broad (first level(L1), one-digit) and narrower (second level(L2), two-digit) levels. We create a 3-level hierarchical taxonomy by manually linking Field-of-Science fields of the sciencemetrix Journal classification to the OECD/FORD level-2 fields. To facilitate a more fine-grained analysis, we extend the aforementioned Field-of-Science taxonomy to level-4 and level-5 fields by employing a pipeline of AI techniques. We evaluate the coherence and the coverage of the Field-of-Science fields for the two additional levels based on synthesis scientific publications in two case studies, in the knowledge domains of Energy and Artificial Intelligence. Our results showcase that the proposed automatically generated Field-of-Science taxonomy captures the dynamics of the two research areas encompassing the underlying structure and the emerging scientific developments.

## 1. Introduction

With the rapid growth of scientific knowledge and literature, a variety of bibliographic databases have been developed to help manage and organize this information. They provide different perspectives and cover a wide range of research areas and include Microsoft Academic Graph (Kuansan et al., 2020) (discontinued), Scopus (Baas et al., 2020), Web of Science (Birkle et al., 2020), Semantic Scholar, Crossref (Howells, 2006), OpenCitations (Peroni and Shotton, 2020), OpenAIRE (Manghi et al., 2019), Dimensions (Herzog et al., 2020), ScienceDirect^1^, and specialized databases such as PubMed^2^ and the Computer Science Ontology (Salatino et al., 2018). Those databases offer a wealth of information, including abstracts, citations, and full-text articles, making it easier for researchers to locate and access relevant literature. Additionally, most of them also follow specific classification systems of science. In the field of bibliometric and scientometric research, these classification systems play a crucial role. They are used to categorize venues (journals or conferences) or individual publications into specific research areas, making it easier to conduct literature searches, analyze the structure and development of scientific disciplines, conduct bibliometric evaluations, and thus, are an important tool in understanding the complex landscape of scientific research and its evolution over time.

In recent years, the scientometrics community has been shifting its focus from classifying research at the venue level to classifying it at the publication level (Eykens et al., 2021), (Hoppe et al., 2021), (Kandimalla et al., 2021), (Waltman and van Eck, 2012), (Rivest et al., 2021). To do so they train and employ machine learning systems that classify the publications according to Field-of-Science (FoS) taxonomies. These taxonomies mostly organize scientific fields hierarchically, where the top levels represent disciplines and broad scientific fields like *engineering and technology* and the lower levels represent more granular research areas like *energy*. Examples of FoS taxonomies are the: All Science Journal Classification (ASJC) System, Frascati Manual Classification (OECD, 2015), WoS Categories and Subject Areas^3^, Scopus Subject Areas^4^, European Science Vocabulary (EuroSciVoc)^5^, Microsoft Academic Graph Concepts and the SciNoBo FoS taxonomy proposed in our previous work (Gialitsis et al., 2022). However, many taxonomies are either domain-specific, contain few-levels of granularity and require expert knowledge and manual work to maintain and curate them. Microsoft Academic Graph (hereinafter mag) was one instance where all of the intricacies that accompany the FoS taxonomies were automated. Nonetheless, mag is discontinued and many classification algorithms using it are now suffering from the lack of updates in the taxonomy.

In this work, we propose a novel approach to extend the three-level FoS taxonomy (L1-L3) and AI/ML classifier of our previous work, scinobo. The taxonomy is based on the OECD fields of research and development (FORD) classification and the FoS fields of the journal classification of sciencemetrix. Our approach combines community detection and topic modeling techniques to dynamically extend the taxonomy to three additional levels (L4-L6). By utilizing the classifier of scinobo, we classify millions of publications and extract communities of venues focused on specific subfields, which are considered the Level 4 FoS fields. By analyzing these communities, we uncover the specific research questions they address, and, by employing topic modeling techniques, we discover the latent topics in each community of publications, which are considered the Level 5 FoS and the top n-grams of these topics are considered the Level 6 FoS. Our approach can provide an accurate, up-to-date hierarchical FoS taxonomy of scientific publications and a classification algorithm capable of assigning these FoS to publications. Furthermore, it can help researchers and practitioners in the bibliometrics and scientometrics community to better understand the structure and development of different scientific fields, and to identify emerging research areas, through its dynamic nature. Apart from being able to power search engines and scientific literature tools, the proposed approach can also be useful in Science, Technology and Innovation (STI) policy-making through identifying and tracking the development of key research areas, and for allocating resources for research and development in a more informed and strategic way.

The rest of this paper is structured as follows: In Section 2, we start by revising our previous work of scinobo, in which we built upon the proposed work. Furthermore, we describe the datasets and datasources used for the extension of the FoS taxonomy of scinobo. We provide detailed descriptions of how we created the additional levels of our taxonomy, namely Level 4, Level 5 and Level 6. We formulate classification algorithms to enable us to classify publications in these extended levels and finally we propose an automatic way of also providing labels for the discovered FoS fields of Levels 4 & 5. In Section 3, we describe our experiments in two knowledge domains, Artificial Intelligence and Energy, showcasing preliminary results and samples of our newly extracted FoS fields. More results are available at the Supplementary material. Finally, Section 4 concludes the paper, summarizing the findings, discussing where we stand in accordance to previous work and states our future work.

## 2. Methods

scinobo encompasses a Graph integrating metadata and publication bibliometric information and an Artificial Intelligence (AI)/ Machine Learning (ML) classification system assigning FoS fields to scientific publications according to a predefined FoS taxonomy. In this section, we describe in detail our novel hybrid approach which employs Neural Topic Modeling and Community Detection techniques to dynamically expand the FoS taxonomy currently used by scinobo as the backbone in automatic publication-level FoS classifiers. Additionally, we describe the data used in our experiments and the proposed methodology to create inference mechanisms in the newer more granular levels of our FoS taxonomy.

More concretely, in Section 2.1 we revise our previous work regarding scinobo. scinobo connects venues (journals/conferences) and publications by building a multilayer network (graph) where venues are represented as nodes and the edges between venues reflect the citing-cited relationships within their respective publications. Section 2.2.1 describes the datasources and datasets used and Section 2.2.2 describes the collection and preprocessing steps. Section 2.2.3 outlines in detail the steps followed for generating the Level 4 FoS fields. Venue-to-Venue graphs specific to each Level 3 FoS are created. Edges are created only if both the published venue and the citing or cited venue belong to the list with the most representative venues under the respective Level 3 field. The weight of the edges is based on the number of times a venue has cited another venue or has been cited by another venue. The goal is to create strongly interconnected communities of venues under each Level 3 field through community detection. Section 2.2.4 builds on top of the previous section and describes the approach developed for assigning Level 4 FoS to publications. Furthermore, Section 2.2.5, describes the process of identifying Level 5 FoS fields by creating publication-to-publication graphs for each Level 4 FoS category, detecting communities of publications using the Louvain algorithm, and employing Neural Topic Modeling to discover the latent topics of each Level 5 community of publications. Section 2.2.6 reports that the Level 6 FoS are the n-grams generated from the topic modeling applied in the previous section and 2.2.7 details the inference mechanism at the Level 5 FoS. Finally, Section 2.3 explains the steps adopted to automatically annotate the Levels 4 and 5 FoS. In 2.3.1, we provide a definition regarding synthesis publications which are used in the annotation process of Level 4 FoS. In the next section (Section 2.3.2) we outline the process of providing annotations for Level 4 through the use of Wikipedia and in the last section (Section 2.3.3), we explain the use of large language models in the annotation of Level 5 FoS.

### 2.1. SCINOBO: Field of Science taxonomy

Current methods for classifying fields of study (FoS) have significant challenges when it comes to handling multidisciplinarity, both at the venue (conference/journals) and at the publication level. Most of these methods rely heavily on text-based information, which can be subject to changes in language and discourse norms in specific fields. Furthermore, many of these approaches are limited to specific disciplines or lack the ability to generalize across fields. Additionally, the hierarchical relationships between FoS fields are often not taken into account. scinobo on the other hand assumes that publications primarily cite other publications with similar themes. We connect venues (journals/conferences) and publications by building a multilayer network (graph) where venues are represented as nodes and the edges between venues reflect the citing-cited relationships within their respective publications. The scinobo algorithm classifies a publication *P* into one or more FoS fields based on the venues of the publications that *P* references (out-citations) and the venues of the publications that cited *P* (in-citations). As a result, scinobo is able to classify publications with minimal metadata, using only journal or conference names and citing information.

The FoS taxonomy, used as our classification scheme is underpinned by the OECD disciplines/fields of research and development (FORD) classification scheme, developed in the framework of the Frascati Manual and used to classify R&D units and resources in broad [first level (L1), one-digit] and narrower [second level (L2), two-digit] knowledge domains based primarily on the R&D subject matter. To facilitate a more fine-grained analysis, we extend the OECD/FORD scheme by manually linking FoS fields of the sciencemetrix^6^ classification scheme to OECD/FORD Level-2 fields, creating a hierarchical 3-layer taxonomy. Table 1 provides statistics of the FoS Taxonomy^7^ and Table 2, provides some examples/labels of the FoS Taxonomy.

**Table 1:** Statistics of the extended OECD/FORD classification scheme.

**Levels of FoS**	**Number of FoS labels**
Level 1	6
Level 2	42
Level 3	174

**Table 2:** Statistics of the extended OECD/FORD classification scheme.

**Level 1**	**Level 2**	**Level 3**
Natural Sciences	Physical Sciences	Optics
Social Sciences	Economics and Business	Economics
Engineering and Technology	Mechanical Engineering	Aerospace & Aeronautics

Furthermore, sciencemetrix classification also provides a list of Journal Classifications. We integrate this seed list, by mapping its journals to scinobo nodes and linking them with the relevant FoS. This mapping represents relationships between venues and FoS and is utilized to classify publications in FoS fields. Initially, a small portion of venues has an FoS at Level-2 and Level-3. By utilizing label propagation , we aim to increase the venue label coverage. With label propagation, we basically propagate information from venues with FoS tags to the rest of the venues that do not have a FoS tag. The approach is similar to a nearest-neighbor classification setting, in that a venue is more likely to have the same FoS as the venues it references the most.^8^

**Figure 1:**
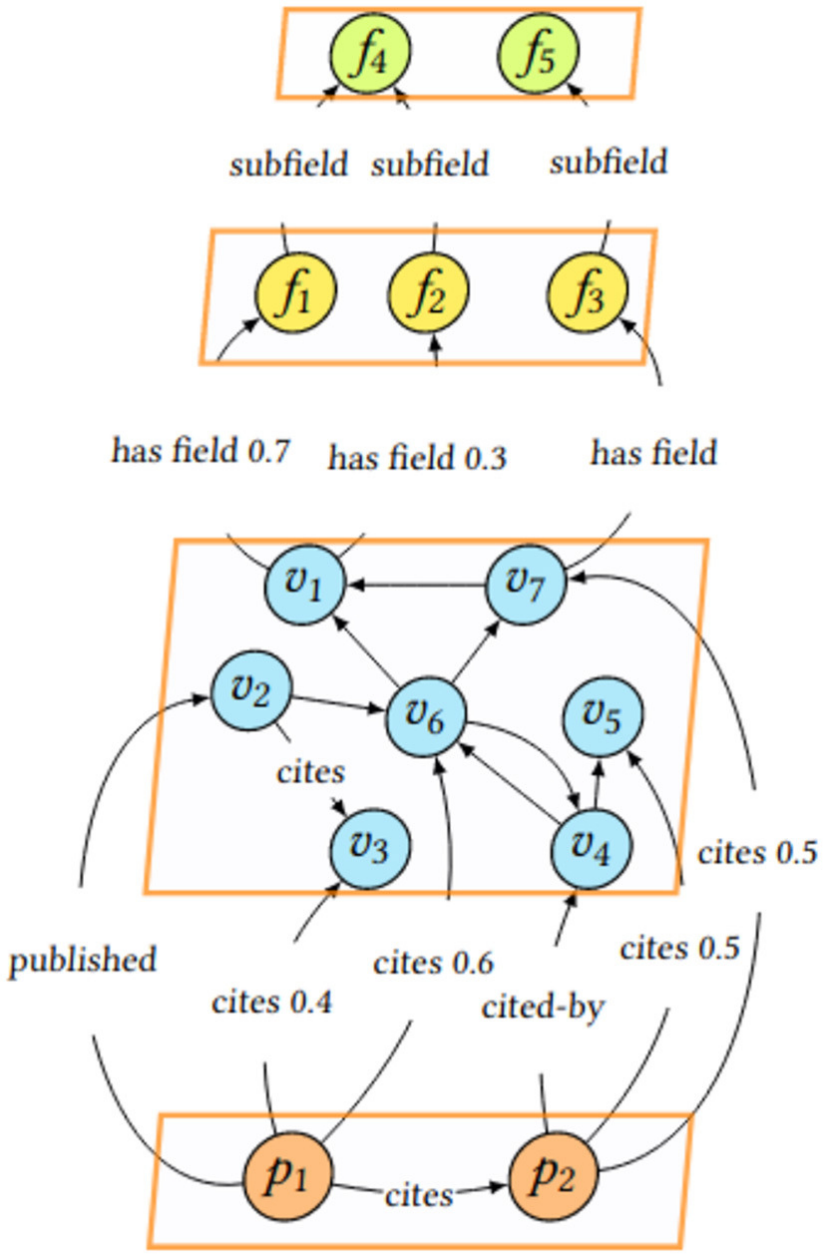
Snapshot of the multilayer graph of scinobo.

A snapshot of the multilayer graph of scinobo is presented in Figure 1. In the Figure, the scientific publications (*p*_*i*_), venues (*v*_*i*_), and the FoS fields (*f*_*i*_) are visible and are connected through different types of edges, like *cites* or *cited-by* for venues and scientific publications and *has field* or *subfield* for venues and FoS fields. The classification step consists of propagating information from the venues linked to FoS fields, to scientific publications.

There exist multiple ways to back-propagate information from the venue level to the publication level depending on the available metadata, as listed below:

- based on the published venue (namely Published-by)
- based on the referenced/cited venues (namely References)
- based on the referenced (cited) and citing venues (namely References+Citations)

**Published-by:** Given a publication *P* and the set of distinct venues (nodes) *P* has been published in (most of the times equal to 1), we draw edges of equal weight from *P* to the venues (nodes). As a result each published venue only contributes the weight it has with its FoS fields. The scores per FoS are aggregated and ranked according to their total weights. The publication is finally classified to the top *T* FoS, where *T* might be fixed or be equal to the number of weights that exceed a user-defined threshold.

**References**: Given a publication *P* and the set of distinct venues it references *K* = {*v*_1_, *v*_2_, ..., *v*_*k*_} we draw edges between *P* and the venues with weight *w*_*P*,_*v*__*i*__ where $v_{i}=\frac{ref\text{\_}v_{i}}{k}$, and *ref*_*v*_*i*_ is the number of referenced publications, published at *v*_*i*_. Similar to the published-by approach, the weights are aggregated and the publications are assigned to the top *T* FoS.

**References+Citations**: This approach is identical to the References one. However, we also take into account the venues that cite publication *P* (*cited-by* edges in Figure 1 if and when available). A methodology originally proposed in the context of one particular field might eventually prove groundbreaking in a completely different field. By incorporating citation venues, scinobo captures cross-domain FoS that would otherwise be missed.

### 2.2. Toward extending the Field of Science taxonomy

The Field-of-Science (FoS) taxonomy described in the previous sections, consists of 3 Levels of granularity. We consider these Levels to be static and not dynamic in the course of time (e.g. Artificial Intelligence and Energy both are well-established research areas at FoS-Level 3). However, to be able to facilitate a more fine-grained analysis and identify emerging and vanishing FoS fields, we need to create a dynamically constructed hierarchical Field-of-Science taxonomy.

#### 2.2.1. Datasources - Datasets

**Microsoft Academic Graph**: Microsoft Academic was a project that leverages the cognitive power of machines to assist researchers in entity exploration of publications and knowledge discovery. Its main outcome was the so-called mag (Kuansan et al., 2020), which is basically a database with millions of records of scientific publications. The heterogeneous graph also contains metadata information such as the authors, affiliations, journals, fields of study, and citation information. An entity disambiguation pipeline is used to do the mapping of those entities. Finally, mag prioritizes magid rather than Digital Object Identifiers (doi), thus some records do not have a doi.

**Crossref**: Crossref is an official digital object identifier (doi) Registration Agency of the International doi Foundation. It is run by the Publishers International Linking Association Inc. and was launched in early 2000 as a cooperative effort among publishers to enable persistent cross-publisher citation linking in online academic journals (Howells, 2006). Crossref contains millions of scientific records and prioritizes the doi identifier.

#### 2.2.2. Data collection and preprocessing

We retrieve all the publications that were published between 2016 − 2021, along with their references and their citations when available. We confine the references in a 10-year window. For every publication, the publishing venue is contained in the metadata. However, this is not the case for the references and citations. As a result, for every publication, we query its references and citations in crossref/mag (by taking the union of the metadata) and we retrieve the original metadata of the reference or the citation. Additionally, we perform a preprocessing step in the publishing venues of the scientific publications, since a considerable challenge is dealing with (i) naming inconsistencies in the reporting of venues in publication references/citations, and (ii) different instances of the same venue. This challenge is particularly prevalent in crossref metadata since the published venue of each publication is being deposited by the members of crossref. Our main goal is to create abbreviations for the names of the venues e.g. the "Empirical Methods in Natural Language Processing" conference should be mapped to EMNLP. Furthermore, different instances of venues should also be mapped to a unique venue abbreviation (e.g. EMNLP 2019, EMNLP 2020, etc. to EMNLP)^9^. In addition, by performing an exploratory analysis of the names of the reported venues, we conclude that most of the abbreviations exist after the character “-” or inside parentheses. Finally, to utilize the aforementioned data for the extension of the Field-of-Science Taxonomy, we must assign the scientific publications to the first three Levels of our taxonomy.

The total number of publications retrieved is 12.492.907 and an instance of a scientific publication from the dataset is presented in the following Table 3.

**Table 3:** Example of the dataset used for the extension of the FoS taxonomy.

**Doi**	**10.1016J.APENERGY.2019.113351**
Published venue	Applied energy
Title	Impacts on industrial scale market deployment of
	advanced biofuels and recycled carbon fuels
	from the EU Renewable Energy Directive II
scinobo Level 1	Engineering and technology
scinobo Level 2	Electrical engineering, electronic engineering,
	Information engineering
scinobo Level 3	Energy

Only the top-predicted FoS triple is presented in the Table. More metadata are available e.g., the abstract. However, they were omitted for simplicity.

#### 2.2.3. Generating Level 4 FoS fields

The intuition behind the proposed approach is that the venues under each Level 3 FoS (e.g., Energy) are creating small communities citing each other. For example, venues that are related to “Renewable Energy Technologies” will cite each other more frequently than venues under other Level 3 FoS or venues that are frequent under other subfields of Energy or “general science” venues. To that end, we utilize the abovementioned dataset to create a Venue-to-Venue graph specific to each Level 3 FoS. Note that we have inferred each publication in the dataset in the first three levels of the FoS Taxonomy. Each scientific publication can be assigned to more than one triple of FoS fields. We keep the most probable (top prediction) one to enforce the constraint of FoS-specific Venue-to-Venue graphs. Furthermore, before creating the graphs, we perform a TF-IDF filtering in the published venues of the publications under each Level 3 FoS field. As a result, for each Level 3 FoS field we end up with a list of venues that are representative for the specific FoS. Additionally, the TF-IDF filtering facilitates removing “general science” venues, such as PlosONE.

After the filtering step, we parse the scientific publications classified into each Level 3 FoS and extract the published venue of the publication and the published venues of its citations and references to create edges for the FoS-specific Venue-to-Venue graph. We create an edge if and only if both the published venue and the citing or cited venue belong to the list with the most representative venues under the respective FoS Level 3 field (creating a closed set of venues), since allowing all cited and citing venues to the venue-to-venue graph will introduce noise. The weight of the edges is the number of times a venue has cited another venue or has been cited by another venue. Our goal is to create strongly interconnected communities of venues under each Level 3 and as a result, we must decide on a threshold weight under which no edge will be created. We consider the threshold weight as a hyperparameter. To tune it, we perform community detection on the FoS-specific venue-to-venue graphs and calculate the average modularity of the generated communities across the Level 3 FoS fields^10^. The algorithm used for community detection is the Louvain algorithm (Blondel et al., 2008). Louvain is an unsupervised algorithm, meaning it does not require beforehand the number of communities nor the size of each community.

The best average modularity was achieved with a threshold weight of 200. We keep the communities with more than one venue and for each community, we keep the top 30^11^ venues (if existing) according to their degree centrality in the respective FoS-specific venue-to-venue graph. The resulting communities of venues are the Level 4 FoS fields under their respective Level 3 FoS field. Recall that our taxonomy has 174 Level 3 FoS fields. Not all of them generate Level 4 FoS fields and the total number of generated Level 4 FoS are 964^12^. Finally, since each Level 3 FoS field generates a number of subfields (Level 4 FoS), annotating them requires time and manual labor. As a result, in 2.3.2, we present an algorithmic approach to automatically annotate and assign Labels to Level 4 FoS.

#### 2.2.4. Inferencing publications at Level 4 FoS fields

Recall that scinobo unifies multiple types of relationships (edges) between entities as well as multiple types of entities under a common framework of operations represented as a multilayer network. As already mentioned, one type of entity in the multilayer network is the venues. The venues are connected to their respective FoS fields at the first three Levels. One key observation, regarding the current inference procedure, described in Gialitsis et al. (2022), is that we first assign a Level 3 FoS to a scientific publication and then follow the hierarchy in the upper Levels. In that way, we enforce the hierarchy in our FoS assignments and we omit errors where an inferred Level 3 FoS does not have a parent in the inferred Level 2 & 1 FoS fields. As a result, we assign to a scientific publication as many triples as the inferred Level 3 FoS fields.

Each generated Level 4 FoS is represented by a community of venues. We add the Level 4 IDs as nodes to the multilayer network and link (i.e., create edges) the venues of each community to their respective Level 4 nodes. Even though we follow the same inference procedure as before (2.1), we do not infer at Level 4 category and then follow the hierarchy as in the Level 3 inference process. As already mentioned some Level 3 fields do not have Level 4s and with a small number of venues having Level 4 fields assigned to them (due to the TF-IDF filtering procedure mentioned in 2.2.3), there is a risk that a lot of scientific publications might not get inferred at all. As a result, we infer the scientific publications in their Level 3 and Level 4 FoS fields, but we filter the Level 4s according to the FoS hierarchy.

#### 2.2.5. Generating Level 5 FoS fields

The Level 5 FoS fields are subfields of the Level 4s. We consider the Level 4 FoS fields as well-established research fields, e.g. Renewable Energy or Natural Language Processing, and Level 5 as evolving research areas with new ones emerging and others vanishing. To delve into the evolving research fields, we must investigate the publications under each Level 4 (community). We retrieve the scientific publications in our dataset (2.2.1) according to their published venue under each Level 4. Even though a scientific publication is published in a venue under Level 4 of Renewable Energy, it could belong to a different Level 4 FoS field according to its citations and references. As a result, we infer all the scientific publications to their respective Level 4 FoS and keep from the predictions the most probable Level 4. We end up with a set of scientific publications that belong to their respective Level 4s with great certainty.

**Figure 2:**
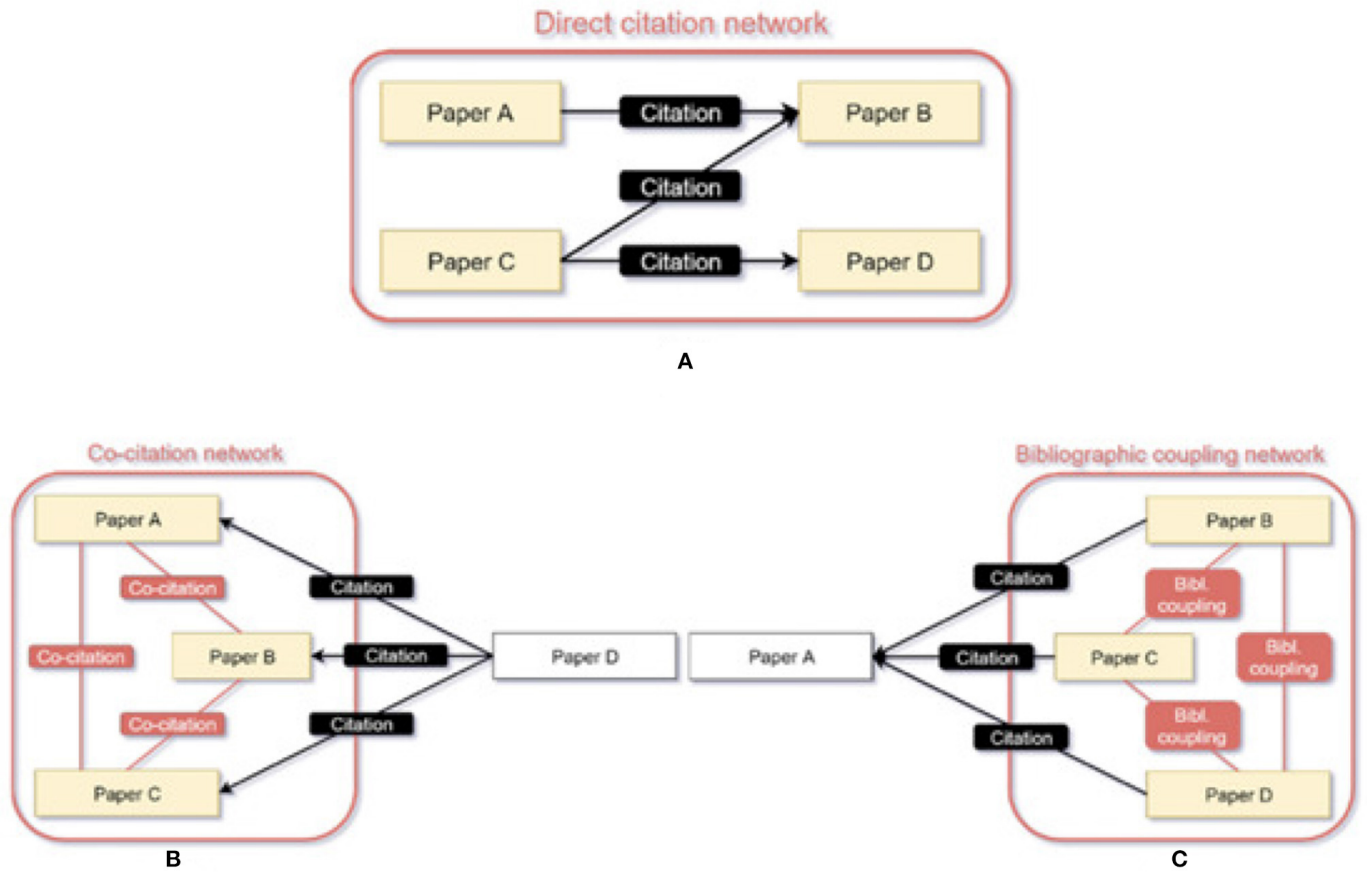
Visual explanation of direct citation **(A)**, co-citation **(B)** and bibliographic coupling **(C)**.

To identify the Level 5 FoS fields, we must first discover the underlying communities created from the scientific publications under each Level 4. Based on the assumption that a scientific publication mostly cites thematically related publications, we can bridge the publications by constructing publication-to-publication graphs for each one of the Level 4 FoS categories, according to their citations and references. The graphs can be created by either employing: *direct citation, bibliographic coupling*, or *co-citation*. Figure 2, originates from Kleminski et al. (2022) and provides a visual explanation of the different approaches applied to create a publication-to-publication citation graph.

Subfigure (a) describes the Direct citation networks, in which Paper B is cited by paper A (has been placed in the reference list of paper A), hence the two are connected by an edge in a directed network. In co-citation (Small, 1973), Paper D cites papers A, B, and C. Respective paper pairs (A and B, A and C, B and C) are thus in mutual relationships in the undirected co-citation network. Finally, in bibliographic coupling (Kessler, 1963), Paper A is cited by papers B, C, and D. Respective paper links (B and C, B and D, C and D) form relationships that are part of the undirected bibliographic coupling network. To create the graphs under each Level 4 FoS category, we utilize the direct citation approach. We employ direct citation because we would like to create a closed set of publications citing each other, as it was described regarding the venues in Section 2.2.3. By using either co-citation or bibliographic coupling, scientific publications not published in the community venues under a specific Level 4 FoS would be introduced. After creating the publication-to-publication graphs, we can employ the same community detection algorithm (Louvain), to generate the communities of publications representing now a Level 5 FoS (an evolving research field). Note, that now we do not have to tune a threshold weight as we did in Section 2.2.3, since the weight of the edges f is either {1, 0}, where 1 indicates a connection between the two publications and 0 indicates no connection.

The main fundamental difference between the generation of Level 4 FoS fields and the Level 5s is that each Level 4 can be represented by a community of venues, which can be interpreted by an expert. On the other hand, each Level 5 FoS field is represented by hundreds to thousands of scientific publications, making it inherently difficult for experts to interpret them. As a result, we employ Topic Modeling and more specifically Neural Topic Modeling, to discover the latent topics of each Level 5 community of publications. Neural Topic Modeling differs from classic topic modeling techniques like Latent Dirichlet Allocation in that it utilizes embedding vectors and Deep Learning techniques to discover latent topics.

We make use of BERTopic (Grootendorst, 2022), which generates document embeddings with pre-trained transformer-based language models, clusters those embeddings, and finally, generates topic representations with the class-based TF-IDF procedure. We use BERTopic, to make use of the contextual information of the abstracts of the scientific publications under each community. BERTopic uses sbert (Sentence-BERT) (Reimers and Gurevych, 2019) to extract contextual embeddings for snippets of textual information. sbert, is a modification of the pre-trained BERT network that uses siamese and triplet network structures to derive semantically meaningful sentence embeddings that can be compared using cosine-similarity. The text preprocessing steps follow Cheng et al. (2022) and Chen et al. (2019)^13^. Since the Level 5 communities were created from a publication-to-publication citation graph and a community detection algorithm, they will be closely related to each other and the latent topics will be few. As a result, we limit the number of topics generated from BERTopic to 5. Finally, note that we have a BERTopic model per Level 5 FoS, with the total number of Level 5 FoS equal to 30360.

#### 2.2.6. Generating Level 6 FoS fields

**Figure 3:**
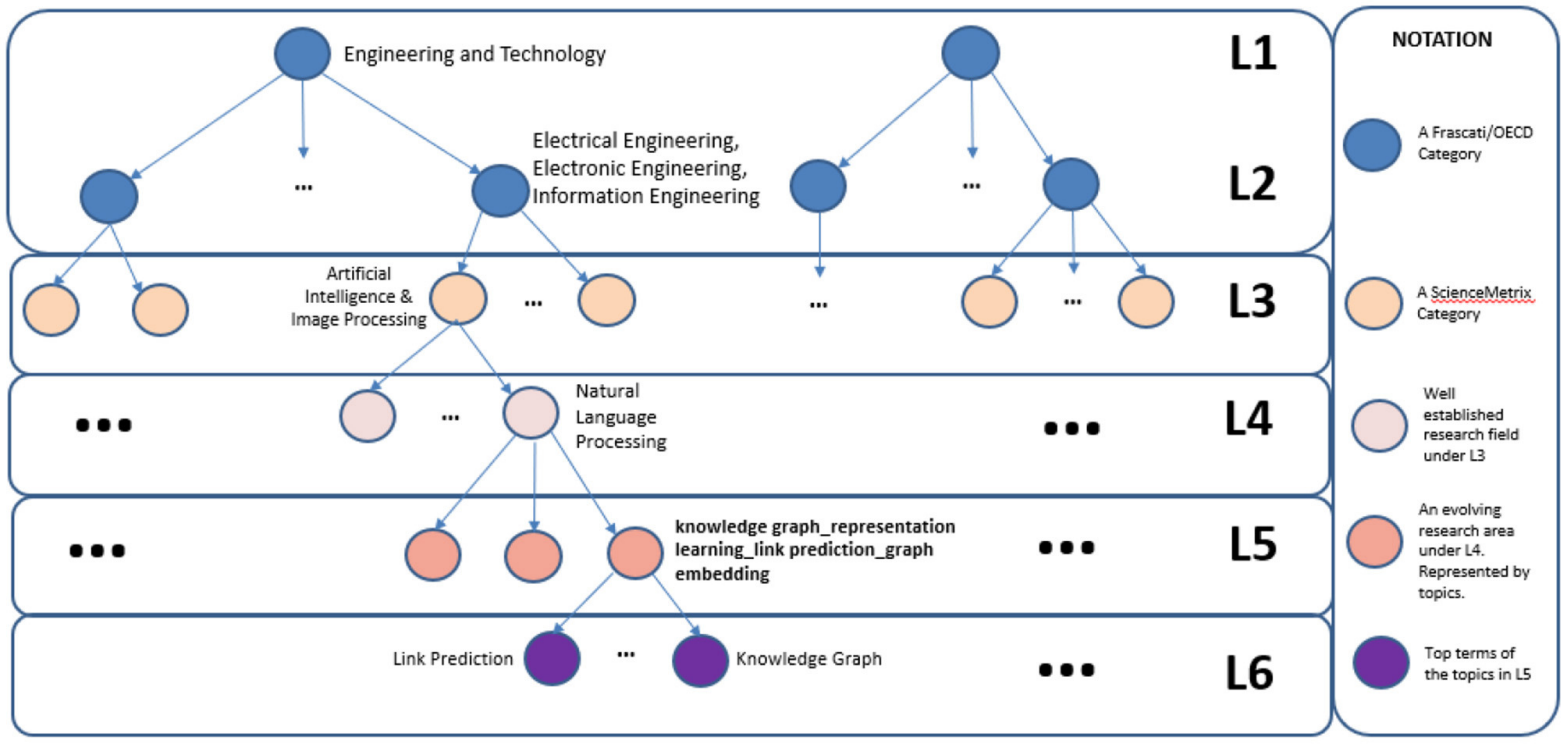
Complete schematic representation of the extended FoS taxonomy.

Regarding Level 6 FoS fields and following previous work Small (1973), Kuansan et al. (2020) (where the FoS fields of the lower levels were terms from Keyterm Extraction algorithms), we consider the words (n-grams) generated from each BERTopic under each Level 5 FoS community as the Level 6 FoS fields. These Level 6 FoS will also be dynamic since they stem from the Level 5 FoS fields. A complete example of the extended FoS Taxonomy can be viewed in Figure 3. We can observe that the first two levels are represented by Frascati/OECD fields, the third Level is a sciencemetrix category and the next three levels are the extended FoS Levels, with Level 4 representing a well-established research field under the Level 3s, e.g. Natural Language Processing, Level 5 FoS fields are evolving research areas under Level 4 and finally the Level 6 FoS are the top terms of the automatically generated topics under each Level 5 FoS category as described above.

#### 2.2.7. Inferencing publications at Level 5 FoS fields

One approach to assigning Level 5 FoS fields to publications, would be to perform topic modeling inference with the trained BERTopic models. However, the trained BERTopic models are as many as the Level 5 fields, which means we would have to load 30360 BERTopic models to infer a single scientific publication. A solution would be to first infer at the Level 4 fields and then for each Level 4 inferred, to only load the specific Level 5 fields under the respective Level 4s. Given the fact that on average each Level 4 has 32 Level 5 FoS fields, this approach still remains computationally inefficient. Given the above, the inference at Level 5, will follow the same principles of the inference mechanisms at the higher levels, with the difference now being, that the FoS fields are not propagated from the venue level to the publication level, but rather from the word level to the publication level. Since each Level 5 is represented by a set of topics, we can utilize the top words of each topic under each Level 5 to create a fast and lightweight inference mechanism. First, we flatten all the top words of the topics under a specific Level 5, then we consider that those words have co-occurred together, so they should be connected in the inference graph of scinobo. It is worth mentioning that some Level 5 FoS have very few publications in their communities. As a result, BERTopic will not generate any meaningful topics. We remove those Level 5 FoS.

**Figure 4:**
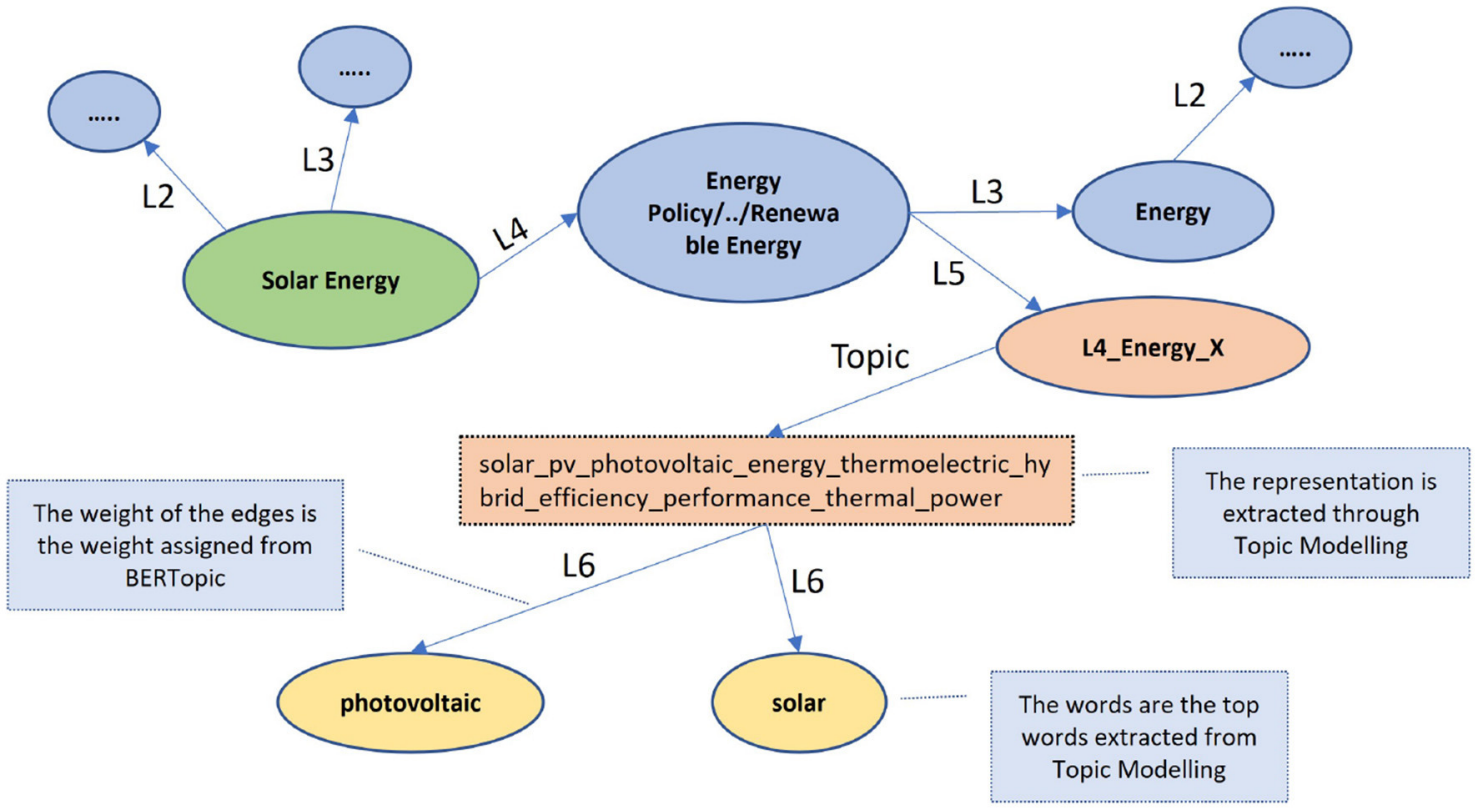
The green node represents a venue, the blue nodes represent FoS fields of all the levels apart from Level 5 and 6. The orange node is a Level 5 (note that it is represented with an ID); and is linked to a topic generated from BERTopic. The yellow nodes are top words under that topic that passed the TF-IDF filtering.

Furthermore, a lot of the top words under each topic are unigrams, pretty common, and do not contribute to differentiating Level 5 FoS fields (e.g. energy). To isolate those words and filter them out of the inference graph, we calculate TF-IDF scores in all the abstracts of our dataset. Finally, we add the words that co-occur to the inference graph of scinobo, drawing edges between them. The weights of these edges are their scores from their respective BERTopic models. We also link the words with their corresponding Level 5 FoS fields in the graph. A snapshot of the inference graph of SciNoBo with all the Level FoS fields is presented in Figure 4.

Given a scientific publication, we retrieve its title and abstract. Since the BERTopic algorithm generates topics with words being from unigrams to trigrams, we generated all the unigrams, bigrams, and trigrams in the concatenation of the title and abstract. To classify a publication *p*, we must map those n-grams to the n-grams in the inference graph. After the mapping, each Level 5 FoS (*L*5_*i*_) is weighted according to the following equation:


(1) $$\begin{array}{l} L5_{i}=\underset{j}{\sum }TF_{w_{j}}\cdot BERTopic\text{\_}score_{w_{j}}, \end{array}$$

where *w* ∈ {1, .., *j*} are the n-grams that are mapped to the Level 5 FoS (*L*5_*i*_) and also exist in the title and abstract of the scientific publication, *TF*_*w*_*j*__ is the term frequency of the j-th n-gram and finally *BERTopic*_*score*_*w*_*j*__ is the BERTopic score of the j-th n-gram. We rank the Level 5 FoS according to the aforementioned equation and return the top-k (k=3) results.

The limitations of this approach are two-fold. Firstly, all the Level 5 FoS are plausible classifications, since we simply map the n-grams from the title and abstract to the inference graph. However, the fact that “solar” exists in the title and abstract should not be a sufficient condition to classify the publication as a Level 5 related to “solar energy”. We must first identify that the publication *p* is related to “Renewable Energy” and then move into assigning the Level 5 FoS. To remedy this, we first infer the publication to its Level 4 FoS fields and inherently boost the information gain of n-grams like “solar” (hierarchy constraint). Secondly, to avoid inferring Level 5 FoS fields that only one n-gram from the title and abstract mapped to their nodes in the graph, we add the constraint that we consider valid Level 5 FoS only the ones that have more than one n-gram in the title and abstract (co-occurrence constraint). Finally, the Level 6 FoS fields are the n-grams (concepts) that led to the inference of the respective Level 5 FoS.

### 2.3. Assigning labels to Level 4 and Level 5 FoS

Manually annotating the newly generated Levels is not feasible. Recall that we have 964 Level 4 FoS fields that are associated with communities of venues. To manually annotate the Level 4s would require expert knowledge in each of the scientific fields and in-depth knowledge of the venues associated with each field. Furthermore, we have 30360 Level 5 FoS fields. Each Level 5 is associated with a certain number of topics, automatically generated from BERTopic. Again, manually annotating them would require a lot of human resources in terms of time and expert knowledge.

#### 2.3.1. Synthesis publications

According to previous work Klavans and Boyack (2017), Sjgrde and Ahlgren (2020), publications with at least 100 references can be considered as gold standards for scientific fields, and the concentration of their references can be used to evaluate and compare different methods for creating scientific taxonomies. In other words, publications with more than 100 references are usually: literature reviews and surveys, and in general, they try to sum up a scientific field. There is a strong rationale for this proposal, both from a historical and a contemporary perspective. From a historical perspective, it has long been recognized that synthesis publications play a special role in the fabric of science. They serve both contemporary and historical functions, informing researchers about current research and weaving it into a broader context. Those publications are also known to have more references (hence the threshold of 100 references) and to be more highly cited, on average, than articles reporting on original research. In fact, it has been suggested (Price and Gursey, 1975) that a synthesis (review) publication should be published after every 30–40 publications in order to summarize earlier research that may have been overlooked or “lost from sight behind the research front”. Guidelines for writing literature reviews often give similar advice, recommending that the synthesis publication should be kept focused but of broad interest.

Our goal is to retrieve enough synthesis publications for each of the Level 3 FoS fields and utilize those publications to automatically extract labels (names) for Level 4 and 5 FoS and also evaluate them. We do not want to annotate Level 6 FoS since these are concepts capturing the dynamics of emerging topics. Examples of synthesis publications in the domains of Energy and Artificial Intelligence are presented in the following Table 4.

**Table 4:** Examples of Level 3 FoS and the corresponding synthesis publications.

**Field of study**	**Title of synthesis publication**
Energy	Pathways of lignocellulosic biomass conversion to renewable fuels
Energy	Hybrid renewable energy systems for desalination
Artificial Intelligence	Applying Natural Language Processing and Hierarchical Machine Learning
	Approaches to Text Difficulty Classification
Artificial Intelligence	Computer vision-based object recognition
	for the visually impaired in an indoors environment: a survey

By exploring the titles of the sample of synthesis publications in the above Table 4, we can observe that the energy synthesis publications summarize technologies and approaches regarding “Renewable Energy”. In addition, the other publications regarding artificial intelligence, sum up aspects/topics in the domains of “Natural Language Processing” and “Computer Vision”. Based on the adopted definition of the synthesis publications, we kept all the scientific publications with more than 100 references. Additionally, we inferred the publications to all the first three Levels of our FoS Taxonomy, keeping for each publication the most probable Level 3 FoS prediction. In total 118557 synthesis publications were extracted^14^.

#### 2.3.2. Labeling Level 4 FoS

We use the titles of the synthesis publications to generate Nominal Chunks (hereinafter NCs). We start by preprocessing the titles, with standard text preprocessing techniques (e.g. Lemmatization, POS tagging, and stopword removal). The intuition behind the NC extraction is that those NCs, most probably, will contain the name of the scientific fields, which the synthesis publications aimed to summarize. One aspect of this approach that requires attention, is that the title might also include technologies and very granular fields that would belong to the Level 5 FoS fields. We perform the same text preprocessing in the section titles of the synthesis publications and filter out the common NCs^15^. Furthermore, we utilize the inference mechanism described in Section 2.2.4 to infer the synthesis publications at Level 4. As a result, we can create a mapping between NCs and Level 4 FoS fields. One approach to naming the Level 4s would be to sort their respective NCs according to term frequency and use the top-ranked NCs. However, that would lead to unigram words occupying the most frequent ranks, with those words also having overlaps among Level 4s.

To alleviate this, we would like to aggregate the semantically similar NCs, create clusters, and then assign to each Level 4 its most frequent cluster of NCs. We employ Agglomerative Clustering (Jain et al., 1999, Kaufman and Rousseeuw, 2009). Agglomerative clustering is a bottom-up hierarchical clustering method. It starts by treating each data point as a separate cluster and then merges the most similar clusters together until a desired number of clusters is achieved. There are several different measures that can be used to determine the similarity between two clusters, such as the distance between the centroids of the clusters, the average distance between all pairs of points in the two clusters, or the maximum distance between any two points in the two clusters. Since we are dealing with textual data, we extract embedding vectors for each NC and then use the “Cosine Similarity” as the distance metric in the algorithm. Furthermore, for calculating the similarity between two clusters we use the average distance of all pairs of points in the two clusters. If that average distance is greater than a predefined threshold, then the clusters are merged. We consider the predefined threshold as a hyperparameter and we exploit sbert^16^ for generating the embedding vectors for the NCs.

To tune the threshold we calculate the coherence score metric borrowed from Topic Modeling research. In topic modeling, coherence is a measure of how semantically related the words within a given topic are. A topic with high coherence will have words that are more closely related to one another, while a topic with low coherence will have words that are less related. However, attention is needed when comparing and tuning with coherence scores, since a high coherence score does not always mean good and interpretable topics. We consider as topics the cluster of the extracted NCs and we calculate the embedding coherence score, which is defined as follows:

**Intra-topic-similarity:** The similarity of NCs in the same topic. We calculate the average similarity between all pairs of the NCs within each cluster to measure the intra-topic-similarity.

**Inter-topic-similarity:** The similarity of NCs across different topics. We calculate the average similarity between the NCs from two different topics to measure the inter-topic-similarity.

The similarity between two NCs is defined as the cosine similarity of their sbert embedding vectors and the coherence score is calculated as follows:


(2) $$\begin{array}{ll} C_{embedding}\left(t_{i},t_{j}\right)\\ = & \frac{\frac{{\text{INTRA-TOPIC-SIMILARITY}}_{t_{i}}+{\text{INTRA-TOPIC-SIMILARITY}}_{t_{j}}}{2}}{\text{INTER-TOPIC-SIMILARITY}\left(t_{i},t_{j}\right)} \end{array}$$

Note that we want to minimize inter-topic-similarity and also that cosine similarity ranges between [-1, 1]. To avoid negative numbers and invalid fractions, we floor inter-topic-similarity to a very small positive number when it is negative. Since we have to assign labels to Levels 4s and 5s, we need to tune the NCs for every Level 3, whose lower levels we try to annotate. The possible values of the threshold per Level 3 are the values between: [0, 1] with a step of 0.1.

After the tuning procedure for the cluster of NCs is over, we assign to each Level 4 FoS, its most frequent cluster of NCs^17^. To provide a name for each cluster, we utilize the knowledge base of Wikipedia. The motivation behind this choice is that NCs that relate to fields, disciplines, and topics of the greater body of human knowledge can be found in knowledge repositories (Kleminski et al., 2022). Wikipedia's English section is a vast and regularly updated source of knowledge, making it a suitable reference point. The aim of extracting names for the NC clusters using Wikipedia is to create a scheme that is unbiased, as it is maintained by a large number of contributors and supported by verifiable sources. This design choice is not without precedence, since many existing approaches utilize Wikipedia for keyword extraction (Qureshi et al., 2012), (Wang et al., 2015) or even modifying algorithms like TextRank (Li and Zhao, 2016), (Yu and Ng, 2018).

To achieve this Level 4 annotation, we make use of Wikipedia API, which enables us to retrieve Wikipedia pages related to each NC. For each NC in the assigned clusters of each Level 4, we retrieve the top 5 Wikipedia pages. Furthermore, we also retrieve the Categories associated with those pages. By filtering the categories to the ones that belong to the Scientific Disciplines of Wikipedia we end up with a list of categories for each cluster of NCs. The top-3 (if available) most frequent categories are assigned as the name of the cluster and consequently the name of Level 4.

#### 2.3.3. Labeling Level 5 FoS

We have defined Level 4 FoS fields as well-established research fields under the Level 3 FoS fields. However, the same does not apply to Level 5 FoS, since they represent evolving research areas. They stem from performing community detection in publications under a specific Level 4 and then applying Topic Modeling to generate well-defined topics describing them. They are valid Level 5 as far as they have enough scientific publications to be discovered by a Topic Modeling technique. New ones will emerge over the course of time and the ones that stop receiving publications will steadily decline. As a result, "the assumption of Wikipedia general completeness fails in regards to emerging and not fully established concepts and fields of study. If a given direction of scientific inquiry does not have a sizable body of literature backing it up and is not widely recognized, it might not have a page associated with itself" (Kleminski et al., 2022). A common approach to assigning labels to topics derived from Topic Modeling is to use word embeddings and try to exploit the top-N words of the topics to create a human-readable label. Motivated by those approaches and the recent advancements in prompt learning and generative modeling, we utilize GPT-3 (Brown et al., 2020) to automatically generate a label for each one of the topics extracted with BERTopic. We consider the most frequent topic under each Level 5 as the topic to represent it and by utilizing the following prompt we automatically generate labels for the Level 5 FoS:

**Input**: *Given the following keyterms:* {Top words of the Topic}
**Prompt**: *Name the subfield of {Level 3* *FoS* *} based on the keyterms:*

The drawback of the aforementioned prompt is that it is possible that GPT-3 returns as output the Level 4 FoS field whose Level 5 FoS we try to label. Consequently, the Level 5 FoS which are labeled with the same name as Level 4 undergo a second round of labeling with a more fine-tuned prompt as defined below:

**Input**: *Given the following keyterms:* {Top words of the Topic}
**Prompt**: *Name the subfield of {Level 4* *FoS* *} based on the keyterms:*

## 3. Experiments and results

AI and Energy are two scientific domains that have seen significant advancements in recent years. AI, which encompasses a wide range of technologies and approaches, is focused on creating intelligent machines that can perform tasks that typically require human intelligence, such as perception, reasoning, and decision-making. Energy, on the other hand, is concerned with the production, distribution, and consumption of energy to meet the needs of society. Furthermore, both of these fields belong to the Level 3 FoS fields in scinobo. We showcase preliminary results, providing Level 4 communities and Level 5 topics (Sections 2.2.3, 2.2.5), tuning results and clusters of NCs (Sections 2.3.2, 2.3.3) and automatically assigning labels at Levels 4 and 5. We provide samples for simplicity and readability reasons, however, extensive results are provided in the supplementary material of this paper. The number of synthesis publications used in the experiments is 977 for Energy and 3215 for AI.

### 3.1. Level 4 FoS fields results

Recall that the FoS taxonomy has 174 Level 3 FoS fields. For each one of them, we create a specific venue-to-venue graph, with venues from scientific publications classified to Level 3 FoS. We perform community detection and the resulting communities represent Level 4 FoS. Initially, these communities are associated with an id and a set of venues closely related to each other. Examples of Level 4 FoS fields from the domains of Energy and AI are presented in Table 5.

**Table 5:** Examples of Level 4 FoS fields under the domain of energy and AI.

**Level 4 ID**	**Community of venues**	**Manual annotation**
L4_AI_9	**(“acl”, “naacl”, “tacl”)** (“acm trans asian low resour lang inf process”) (“coling”) (“computational linguistics”) (“emnlp”) (“ijcnlp”) (“int joint conf artif”) (“lang resources evaluation”) (“nat lang eng”) (“national conference on artificial intelligence”)	Natural Language Processing
L4_Energy_11	(“clean techn environ policy”) (“ecol econ”) **(“energy econ”, “energy efficiency”, “energy policy”)** (“energy for sustainable development”) **(“energy research and social science”, “energy research social science”)** (“energy sources part b economics planning and policy”) (“energy strategy reviews”) (“energy sustain dev”) (“environ sci pollut res”) (“front energy res”) (“int j life cycle assess”) (“journal of cleaner production”) (“nat energy”) (“renew sust energ rev”) (“resources policy”) (“waste manag”)	Renewable Energy

The Level 4 FoS fields get an ID as seen in the first column. The second column presents the communities of venues under each Level 4. The third column presents possible interpretations/labels of the communities presented in the second column.

**Figure 5:**
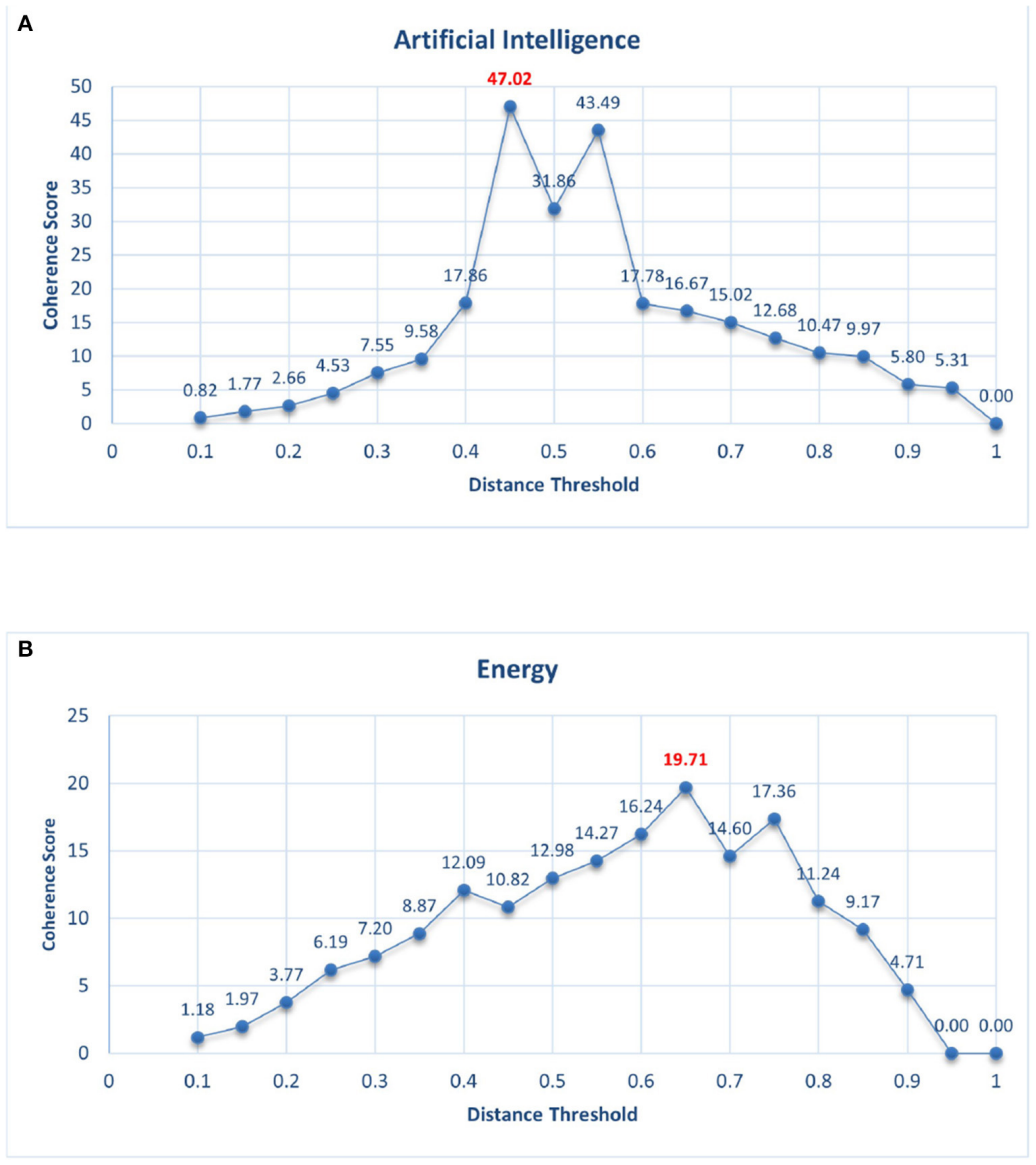
**(A)** Tuning results for AI. **(B)** Tuning results for energy. Coherence scores per distance threshold for the domains of energy and AI when tuning the Level 4 NCs. Best results achieved with distance=0.65 and coherence score=19.71 for energy and distance = 0.45 and coherence score = 47.02 for AI.

The venues in bold under the column “Community of Venues” have been clustered according to their lexical similarity for presentation and readability reasons. Furthermore, the Venue Deduplication process described in Section 2.2.2 might fail to map a Venue name to its abbreviation, as can be seen with *"energy research and social science"* and “*energy research social science”* in the second row. These kinds of errors are attributed to the fact that we extract abbreviations for the venues, not only from the provided metadata in the crossref and mag records but also from applying text processing in the textual information deposited from the members of crossref, as already mentioned. We can observe, that the presented communities are well formed, and if properly interpreted and labeled, can represent real Level 4 FoS under the knowledge domains of Energy and AI. However, as it is also visible, annotating all the Level 4 FoS solely based on their communities of venues can be time-consuming and sometimes impossible since the venues do not always directly indicate the research area they are involved with. The need of developing an automatic way of annotating these fields is evident. Based on the proposed methodology described in Section 2.3.2, we provide in Figure 5 tuning results regarding coherence scores per threshold. As it can be observed, the most coherent NC clusters were achieved with *distance=0.65* and *coherence score=19.71* for Energy and *distance=0.45* and *coherence score=47.02* for AI. In Table 6, examples of NC clusters are presented.

**Table 6:** Example of cluster assignments to Level 4 FoS IDs under Level 3 of energy and AI.

**Level 4 ID**	**Cluster of NCs**
L4_AI_9	“Natural language processing article”, “natural language”, “natural language processing”, “nlp”, “speech language processing”, “computational natural language processing”
L4_AI_4	“Machine vision”, “computer vision”, “large scale visual recognition”, “computer vision technique implication”, “automatic recognition”, “computer vision technique”, “computer vision object recognition”, “automatic identification”, “artificial vision”, “automatic visual detection”,
L4_Energy_11	“Renewable energy urbanization”, “electricity consumption”, “renewable energy production”, “renewable energy development”, “energy pollution growth nexus”, “renewable energy utilization”, “sustainable renewable energy”, “sustainable energy”, “hybrid solar technology”, “clean energy generation”
L4_Energy_10	“Bioenergy farming”, “high grade solid biofuel”, “biomass pyrolysis”, “current biofuel production”, “bioenergy knowledge perception”, “bioenergy generation”, “bioenergy resource assessment”, “biofuel crop”, “sustainable biomass”, “algal biofuel generation”

Some clusters have more NCs, however for readability reasons, we have randomly sampled, if available, 10 NCs per cluster.

The presented clusters are the most frequent for each of Level 4 in the Table. We notice that the clusters are coherent, the NCs are semantically similar, and are also relevant and eligible for the Level 4 FoS fields. One approach would be to use these NC clusters as the labels of their respective Level 4s. However, the clusters might contain hundreds of NCs or even specialized NCs that are difficult to interpret. By utilizing, the Wikipedia approach described in 2.3.2, we can search the Wikipedia pages of the NCs and retrieve the top 3 most frequent scientific Wikipedia categories per Level 4. The results of the proposed Wikipedia approach for Level 4 FoS in Table 6 are presented in Table 7. Results regarding all the Level 4 FoS of Energy and AI are provided in the Supplementary material.

**Table 7:** Results of the Wikipedia approach for assigning names to Level 4 FoS.

**Level 4 ID**	**Wikipedia assigned annotation**	**GPT-3 assigned annotation**
L4_AI_9	Natural language processing/computational linguistics	Computational natural language processing
L4_AI_4	Applications of computer vision/computer vision/image processing	Computer vision
L4_Energy_11	Energy policy/renewable energy commercialization/Renewable energy	Renewable energy science
L4_Energy_10	Biomass/biofuels/bioenergy	Bioenergy science

Results are also presented using prompts with GPT-3 to further evaluate the results. The top-3 (if available) returned categories of the Wikipedia approach are shown.

All the Wikipedia assigned names in Table 7 are the research areas from which the NCs in Table 6 stem, encompassing the underlying structure of the Level 4 FoS fields. To further validate the results, we also experimented with GPT-3 automatically assigning a label to each Level 4, given its assigned cluster of NCs. The prompt utilized is as follows:

**Input**: *Given the following keyterms:* {Cluster of NCs}
**Prompt**: *Name the field of science based on the keyterms:*

### 3.2. Level 5 FoS fields results

To better understand the automatic annotation at Level 5 FoS with the proposed approach described in Section 2.3.3, we present some qualitative results. Table 8 presents five discrete Level 5 FoS under the Level 4 FoS presented in Table 7. We observe that all the Level 5 presented encapsulate technologies, approaches, and topics of their respective Level 4 FoS. Note that similar Level 5 FoS might occur under different Level 4s. For example, with reference to the Supplementary material, *Deep Learning* can also be seen under *Natural Language Processing* and under *Computer Vision* as well. Furthermore, *Renewable Energy Technologies* can also be seen under Level 4 of *Bioenergy* and *Renewable Energy*, since they are closely related. Additionally, Table 9 presents the most frequent topic descriptors associated with each Level 5 FoS presented in Table 8. Observe that the topics are descriptive enough for a generative model (GPT-3) to infer the field described. Finally, duplicate topic words can exist between topic descriptors, validating the design choice of the inference mechanism at Level 5, where the procedure first classifies at Level 4 and then at Level 5 FoS.

**Table 8:** Automatically extracted annotations for Level 5 FoS in the domains of energy and AI.

**Level 4 FoS name**	**GPT-3 assigned names of Level 5 FoS**
Natural language processingComputational linguistics	Name entity recognition (ner) Neural machine translation (nmt) Argument mining Dependency parsing Event extraction and detection
Applications of computer vision/Computer visionImage processing	Pedestrian detection Action recognition Video object segmentation Image denoising Pavement crack detection
Biomass/Biofuels/Bioenergy	Biomass torrefaction Bioenergy pyrolysis Biomass pretreatment Pyrolysis Biodiesel production technology
Energy policy/Renewable energy commercialization/Renewable energy	Hydropower energy Solar photovoltaic energy Carbon emission reduction Municipal solid waste management Renewable energy policy and planning

**Table 9:** Most frequent topic descriptors associated with each Level 5 annotated.

**GPT-3 assigned names of various Level 5 FoS**	**Most frequent topic descriptor per Level 5**
Name entity recognition (ner) Neural machine translation (nmt) Argument mining Dependency parsing Event extraction and detection	Entity/name/name entity/ner/entity recognition Translation/machine/machine translation/language/nmt Argument/argumentation/mining/annotation/task/argumentative Parse/dependency/parser/tree/language Event/extraction/argument/event extraction/detection
Pedestrian detection Action recognition Video object segmentation Image denoising Pavement crack detection	Detection/image/pedestrian/propose/network Action/video/temporal/network/feature/action recognition Segmentation/video/object/object segmentation/video object Noise/image/denoise/denoising/image denoise Crack/detection/crack detection/pavement/network
Biomass torrefaction Bioenergy pyrolysis Biomass pretreatment Pyrolysis Biodiesel production technology	Torrefaction/biomass/torrefy/energy/temperature Pyrolysis/biomass/yield/lignin/reaction Lignin/high/pretreatment/cellulose/biomass Pyrolysis/microwave/oil/waste/biomass Catalyst/biodiesel/production/biodiesel production/heterogeneous
Hydropower energy Solar photovoltaic energy Carbon emission reduction Municipal solid waste management Renewable energy policy and planning	Hydropower/energy/small/plant/impact Solar/pv/photovoltaic/energy/power Emission/carbon/trading/emission trading/carbon emission Waste/management/solid waste/solid/municipal Energy/policy/emission/climate/renewable

Only 5 words (n-grams) per topic descriptor are visible for presentation reasons.

## 4. Discussion and conclusion

In this work, we build upon our previous system of scinobo, which established a three-level FoS taxonomy and an AI/ML classifier that uses graph-based bibliometric information to classify publications. The FoS taxonomy of scinobo was created by utilizing the two-levels of the OECD fields of research and development (FORD) classification, developed in the framework of the Frascati Manual and the FoS fields of the journal classification of sciencemetrix, linking them together in a three-level hierarchy. These first three levels are used as “seed” FoS fields and facilitate the extension of our taxonomy. To that end, we propose a novel approach combining community detection and topic modeling techniques to dynamically extend our current taxonomy to three additional levels. By utilizing the classifier of scinobo, we classify millions of publications with high confidence scores, creating high quality closed sets of publications per Level 3. By extracting the publishing venue from each of the classified publications and creating venue-to-venue citation graphs, we discover communities of venues, with each community being focused on a specific subfield under Level 3. The intuition here follows a nearest neighbor setting, since venues that cite each other multiple times, most probably address the same *research topics*. The extracted communities are regarded as Level 4 FoS fields in our new dynamic taxonomy and each one is represented by a set of venues. Furthermore, by analyzing these communities and now investigating their publications we uncover the specific *research topics* each community is addressing. The methodology is similar to that used in Level 4 discovery, however we now delve into the relationships of the published scientific literature, creating publication-to-publication graphs and repeating the community detection step. Finally, by employing Topic Modeling techniques we discover the latent topics existing in each community of publications. The top-words associated with the topics are considered to be Level 6 FoS.

Level 4 FoS are well-established research areas. However, Levels 5 & 6 FoS incorporate new emerging fields and topics capturing the dynamics in scientific developments. By periodically updating the publication-to-publication graphs and the topics at Level 5, we discover these emerging fields and topics. Finally, by following previous work in automatically providing labels for topics, we propose two discrete approaches, a Wikipedia-based approach for labeling Level 4 FoS and a language modeling approach for labeling Level 5 FoS.

The design choices of the proposed work are in a way similar to Shen et al. (2018). They start with *seed* FoS and employ a graph link analysis in a nearest neighbor setting on Wikipedia entities to augment and expand the FoS fields in their taxonomy. In relation to that, we also utilize a graph methodology to propagate venue FoS fields to venues that do not have an FoS as described in Section 2.1. Furthermore, Shen et al. describe a classifier using text in an embedding-based fashion which also uses bibliometric information (citations, references and venues) to assign their FoS fields to publications. In contrast, we infer at our first 4 Levels of the SciNoBo taxonomy by exploiting bibliometric information and only utilize textual information in classifying at Levels 5 and 6 FoS. Finally, to create their taxonomy, Shen et al. make use of a co-occurence approach, where if FoS *x* subsumes *y* and if *y* occurs only in a subset of the documents that *x* occurs in, then *x* is the parent of *y*. This comes with some drawbacks since their FoS fields from the second level and onward are Wikipedia entities, containing concepts like *proteins* or even *diseases*. Their FoS fields are not intuitive and their higher levels do not always describe scientific fields of research. As a result, misconceptions like *polycystic kidney disease* (a disease) being the parent of *kidney* (an organ) occur. scinobo on the other hand, adopting a top to bottom approach for creating its dynamic taxonomy, enforces the hierarchy among the different levels and FoS fields. Furthermore, we make sure that Level 4 FoS fields (with Wikipedia 2.3.2) are real scientific fields, by filtering the categories returned from Wikipedia to be scientific categories. Additionally, the prompts used for Level 5 annotation, enforce the generative LLM model to answer in the context of a scientific field. Finally, our Level 6 FoS are concepts extracted from Topic Modeling where relevant FoS (e.g., diseases and organs) are most likely to be discovered.

Our work is not without limitations. Note that when we perform inference at Level 5 FoS fields, we extract from the title and abstract of a publication *P* its n-grams, which we map in our inference graph of scinobo as described in Section 2.2.7. This mapping is performed through string matching. A drawback of this approach is that its recall is low. For example, an n-gram like *building performance simulation* will not map to the inference graph, since only the n-gram of *building simulation* is available. To alleviate this unwanted effect, we can semantically enhance the mapping by exploiting sentence embeddings (SBERT) and performing the matching as semantic search through embedding vectors. Another limitation is the annotation at Level 5. We utilize a generative model to produce a label for a FoS at Level 5. Generative models produce a sentence that best answers the prompt which they were given. This approach might introduce noise, since generative models' responses can lead to *hallucination* by providing non-existent answers or even providing a large sentence as an answer which describes the scientific field we aim to annotate. A solution to remedy this, is to also employ the Wikipedia database to provide annotations for Level 5s. Recall that Wikipedia will not always contain information for emerging FoS, however, these should be kept and described with their most frequent topic. We leave this methodological path as future work.

In future work, we plan to formulate an approach to better model scientific advances. We can divide them into *emerging scientific interdisciplinary* *FoS* ^*SK*^ *fields, emerging scientific Level 5* *FoS* ^*SK*^ *fields* and *emerging scientific topics under Level 5* *FoS* ^*SK*^. *Emerging scientific interdisciplinary* *FoS* ^*SK*^ *fields*, will be based on interdisciplinary research. Interdisciplinary research can be defined as a mode of research by teams or individuals that integrate information, data, techniques, tools, perspectives, concepts, and/or theories from two or more scientific disciplines or bodies of specialized knowledge to advance fundamental understanding or to solve problems whose solutions are beyond the scope of a single discipline. To keep track of *Emerging scientific interdisciplinary* *FoS* ^*SK*^ *fields* we will utilize our Level 3 FoS and track the growth rates of interdisciplinary areas like “*AI and Energy*”. *Emerging scientific Level 5* *FoS* *fields* will be based on our Level 5 FoS fields, where we can define metrics of tracking their growth rate and finally *emerging scientific topics under Level 5* *FoS* ^*SK*^ are the most frequent topics under each Level 5 FoS in which we plan to propose a methodology of periodically updating them utilizing our proposed inference mechanisms and Topic Modeling techniques.

## Data availability statement

The datasets presented in this study can be found in online repositories. The names of the repository/repositories and accession number(s) can be found in the article/Supplementary material.

## Author contributions

HP contributed to the conception and design of the study and reviewed the manuscript. NM evaluated the results. SK and DP performed the data analysis and experiments. SK wrote the first draft of the manuscript. All authors contributed to the article and approved the submitted version.

## Footnotes

^1^For detailed information on ScienceDirect see https://www.sciencedirect.com/.

^2^For detailed information on PubMed see https://www.ncbi.nlm.nih.gov/pubmed/.

^3^For a detailed catalog of WoS categories see https://images.webofknowledge.com/images/help/WOS/hp_research_areas_easca.html.

^4^For a detailed list of Scopus categories see https://service.elsevier.com/app/answers/detail/a_id/15181.

^5^See https://op.europa.eu/el/web/eu-vocabularies/euroscivoc for more information on EuroSciVoc.

^6^For detailed information on ScienceMetrix visit https://science-metrix.com/.

^7^For a comprehensive view of the Field of Science Taxonomy, please refer to the OpenAIRE website.

^8^Refer to our first paper for more information (https://dl.acm.org/doi/abs/10.1145/3487553.3524677).

^9^The following preprocessing was applied to the names of the venues: Removal of latin characters, cardinal, and ordinal numbers, dates, days, months, pre-specified words/phrases (e.g. "speech given", "oral talk" etc.), stopwords, special characters; adding a space character when removing them and normalizing multiple spaces. The same preprocessing procedure is also applied during inference.

^10^The possible values of the threshold weight are: {50, 100, 120, 200, 300, 400}.

^11^We empirically choose 30. However, we can also consider it as a hyperparameter.

^12^Examples of Level 4 FoS fields under the domain of Energy and Artificial Intelligence are presented in the Supplementary material.

^13^We remove English stopwords, remove punctuation, strip multiple whitespaces and lemmatize words using SpAcy. Additionally, we also add to the stopwords, common words used in abstracts that do not offer any information to the algorithm (e.g. “et. al.”, “study”, “review”; the complete list is provided in the supplementary material).

^14^Distribution of the synthesis publications in the Level 3 FoS fields is provided in the Supplementary material.

^15^We can also add a seed list of NCs that we do not want to be filtered from the titles of the synthesis publications.

^16^ https://huggingface.co/sentence-transformers/all-mpnet-base-v2

^17^Calculated as the number of scientific publications classified to Level 4, from which the Noun Chunks stem.
